# Advances in aptamer screening and aptasensors’ detection of heavy metal ions

**DOI:** 10.1186/s12951-021-00914-4

**Published:** 2021-06-01

**Authors:** Wenfei Guo, Chuanxiang Zhang, Tingting Ma, Xueying Liu, Zhu Chen, Song Li, Yan Deng

**Affiliations:** Hunan Key Laboratory of Biomedical Nanomaterials and Devices, Hunan University of Technology, Zhuzhou, 412007 China

**Keywords:** Aptamer, Screening, SELEX, Aptasensor, Heavy metals ion, Detection

## Abstract

Heavy metal pollution has become more and more serious with industrial development and resource exploitation. Because heavy metal ions are difficult to be biodegraded, they accumulate in the human body and cause serious threat to human health. However, the conventional methods to detect heavy metal ions are more strictly to the requirements by detection equipment, sample pretreatment, experimental environment, etc. Aptasensor has the advantages of strong specificity, high sensitivity and simple preparation to detect small molecules, which provides a new direction platform in the detection of heavy metal ions. This paper reviews the selection of aptamers as target for heavy metal ions since the 21th century and aptasensors application for detection of heavy metal ions that were reported in the past five years. Firstly, the selection methods for aptamers with high specificity and high affinity are introduced. Construction methods and research progress on sensor based aptamers as recognition element are also introduced systematically. Finally, the challenges and future opportunities of aptasensors in detecting heavy metal ions are discussed.

**Figure:**
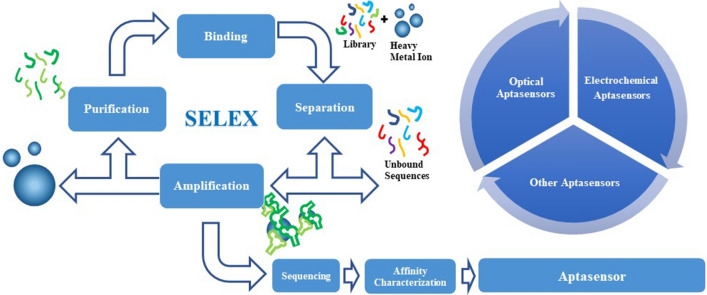

## Background

With development of industry and exploitation of resources, many heavy metal ions enter rivers and soil and cause serious pollution to the environment [1, 2]. Heavy metal ions are difficult to be biodegraded and they can accumulate thousands of times in the human body under biological amplification action of food chain [3]. Moreover, heavy metal ions influence the activity of proteins and enzymes in the human body and accumulate in some organs, which causes chronic poisoning [4, 5]. The pollution of heavy metal ions is seriously harmful to human health, which has aroused great concern in the world [6]. Therefore, it is necessary to develop a rapid, sensitive and reliable method for trace detection of heavy metal ions.

The detection methods for heavy metal ions are various, including traditional methods and novel biosensors [7]. The traditional detection methods include atomic absorption/emission spectrometry, atomic fluorescence spectrometry, inductively coupled plasma mass spectrometry, high performance liquid chromatography and so on [8–12]. These methods can accurately quantify heavy metal ions with high sensitivity but they require professionals for on-site and routine environmental monitoring, and sample pretreatment is complex with expensive equipment, which limits their application [13]. As a novel detection tool, biosensor depends on specific recognition elements such as enzymes/substrates, antigens/antibodies and targets/aptamers [14]. However, enzyme biosensors are sensitive to pH, temperature, pressure and ultraviolet irradiation, and most antibodies are obtained from live animals, which makes them difficult to obtain and expensive to test [15, 16]. Because functional nucleic acid is easy to identify and modify, it is a powerful tool to construct sensor for heavy metal ions detection [17, 18].

Aptamers, also known as artificial antibodies, are single-stranded deoxyribonucleic acid (ssDNA) or ribonucleic acid (RNA) obtained by SELEX (Systematic Evolution of Ligands by Exponential Enrichment) method [19, 20]. Compared with traditional antibodies, aptamers have the advantages of simple preparation, good stability and easy labeling, and higher specificity and affinity to targets [21]. Since 1990, aptamers have been widely used in the detection of cells, viruses, proteins, sugars, antibodies and pesticides [22–24]. However, metal ions have great challenges in aptamer selection and aptamer-based sensor (aptasensor) construction because of their small molecular weight and single binding site [25, 26]. The application of functional nucleic acids as metal aptamers has been studied for 25 years [27]. At present, heavy metal ions, especially Pb^2+^, Cd^2+^ and Hg^+^, are widely used detection targets in aptasensors [28], and the common aptamer sensors include optical aptasensors and electrochemical aptasensors, which show wide range of applications [29, 30]. How to select aptamers with high affinity and construct aptamer sensors for rapid and sensitive detection of heavy metal ions has become a long-term research topic [31–33].

This paper therefore reviews the aptamer selection methods and aptamer sensors construction for detection of heavy metal ions in the past five years, and provides reference for development of aptamer sensors in the research field of heavy metal ions detection.

## Selection of heavy metal ion aptamers

**Fig. 1:**
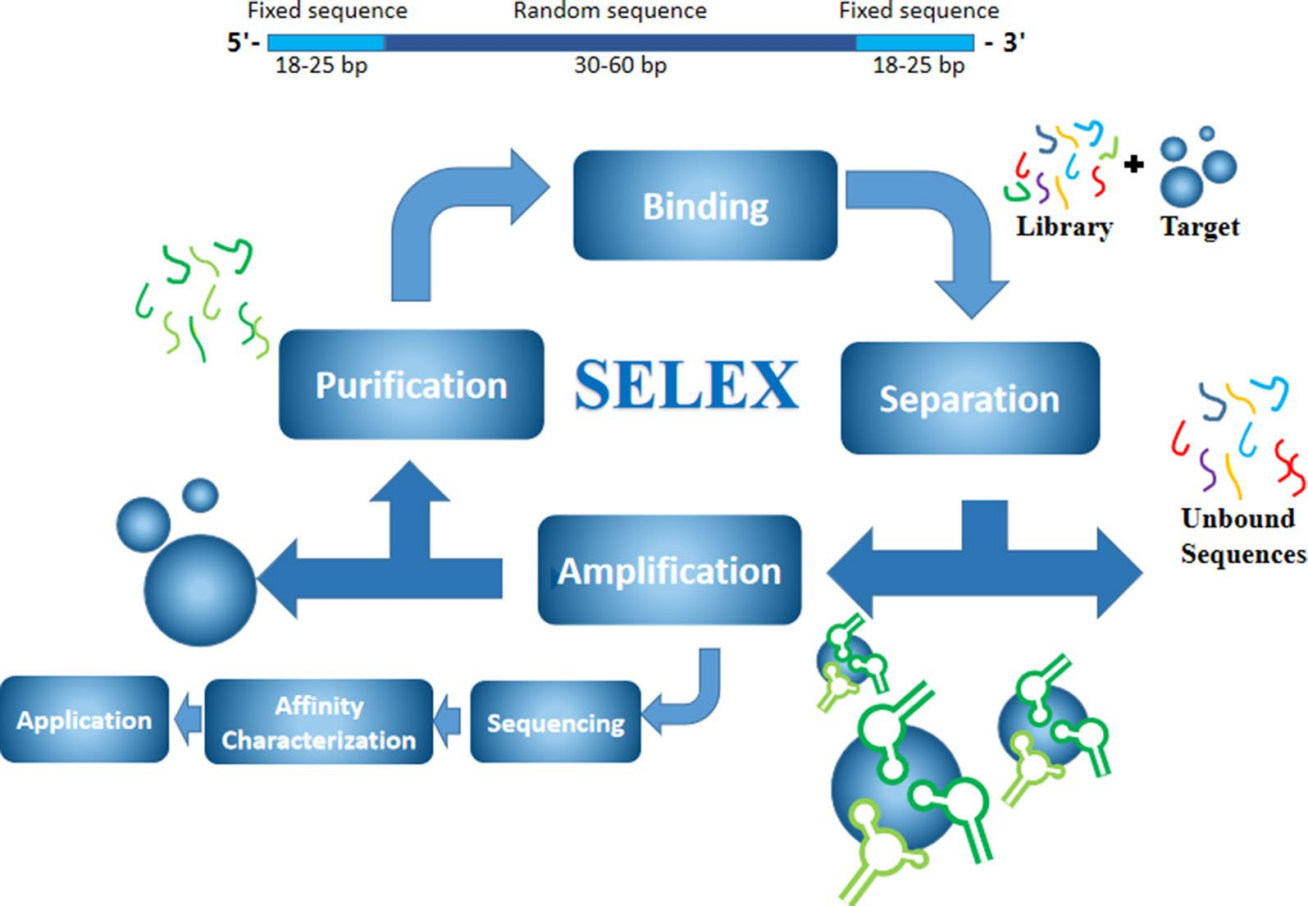
A schematic diagram of SELEX. The initial library contains about 10^14^–10^16^ random oligonucleotides, which is composed of fixed primer sequences with 18–25 bp at both ends and 30–60 bp in the middle. The aptamer is obtained after several rounds of selection, including binding, separation, amplification and purification, and the products are used in the next round of selection

The basic process for SELEX is shown in Fig. 1. The oligonucleotide library is characterized by fixed sequences at both ends and random sequences in the middle of the oligonucleotides [34]. The fixed sequence contains the binding site for primers related to PCR amplification. The random sequence generally contains 30–60 nucleotides which determine different spatial structure for each nucleic acid molecule and ensures the diversity of the library [35]. The SELEX process is mainly repeated in the following four steps: binding, separation, amplification and purification [36]. Oligonucleotides with high affinity to the target are separated from a large of random library, and the purity increases via SELEX process [37]. After multiple cycles of SELEX process, the enrichment of DNA library is monitored to determine the number of cycles that terminate the process [38]. The experimenters further clone and sequence the fragment of SELEX, and then analyze their secondary structure and determine affinity, which finally obtain specific aptamer [39].

Compared with other targets, heavy metal ions have the following advantages and challenges. Aptamer, a kind of polyanion, can attract metal ions by electrostatic interaction [40]. Different aptamer sequences can constitute different three-dimensional structures which have affinities for certain metal ions [41]. Because of simple structure and single binding site for heavy metal ions, the aptamers lack effective recognition sites [42]. Similar structures of different metal ions also make it difficult for aptamers to specifically recognize metal ions in the same group. Moreover, there are challenges in the selection of heavy metal ion aptamers. Heavy metal ions have small molecular weight and small steric hindrance after binding to nucleotide sequence, which will seriously affect their separation in the screening process. Most SELEX methods are difficult to effectively separate highly specific aptamers. Since 1995, Ciesiolka and his colleagues have obtained aptamers of Zn^2+^ by affinity chromatography, extending the target for aptamers to the field of heavy metal ions [43]. Nowadays, the common selection principle for heavy metal ions is to fix the library on the matrix, which includes affinity chromatography-SELEX and Graphene oxide-SELEX [44].

### Affinity chromatography-SELEX

Affinity chromatography-SELEX was first used to select aptamers for organic dyes by Ellington in 1990 [45]. When screening heavy metal ions, agarose or resin is usually filled into the chromatographic column as a solid matrix, and the random library is fixed on the solid substrate under the action of streptavidin–biotin [46]. Heavy metal ions were injected into the affinity chromatography column as mobile phase to incubate and some specific nucleic acid sequences are highly attractive to the target via affinity interaction [47, 48]. The difficulty incubation was improved by reducing the target concentration, and the aptamer with high affinity was obtained [49]. Affinity chromatography has been widely applied to select aptamers for heavy metal ions. Rajendran and Ellington [50] obtained Zn^2+^-specific aptamer beacon by affinity chromatography. The ssDNA sequence was immobilized on the affinity chromatography column and the aptamer was selected by Zn^2+^ solution. From the fourth round, other transition metal ions except zinc were added to negative selection and the Kd was 15 μM after 12 rounds of selection. Wang et al. [51] used the same selection method for target-induced strands release to obtain the Cd^2+^ aptamer Cd-2–1 with high specificity. The conformation of aptamer changes when it binds to Cd^2+^, which shows obvious change on Circular Dichroism (CD) spectral. Chen et al. [52] also used the same method to select aptamers for Pb^2+^. The aptamer Pb-14 s with Kd of 0.76 ± 0.18 μM was obtained after 14 rounds of positive selection and 4 rounds of negative selection. Wu et al. [53] used Cd^2+^ as the target and obtained the aptamer with Kd of 34.5 nM after 11 rounds of positive and negative selection, which was rich in T and G.

Similar to agarose, resin is also an important material for affinity chromatography. Kim et al. [54] established a method for aptamers selection in vitro with an affinity column immobilized arsenic on Affi-Gel 10 resin. The Kd of Ars-3 to detect As (V) and As (III) was 4.95 ± 0.31 and 7.05 ± 0.91 nM. Moreover, Wrzesinskid and Ciesiolka [55] immobilized the RNA library on NTA resin and eluted the library with CoCl_2_ solution. After 15 rounds of selection, the Co^2+^ aptamer with Kd of 1.1 ± 0.15 mM was obtained. Subsequently, they identified the binding sites by Co^2+^-induced cleavage and speculated the chemical properties, such as hardness and coordination structure of metal ions, were important factors affecting aptamers in selection of heavy metal ions.

### Graphene oxide-SELEX

Graphene oxide (GO) is a common separation medium and SELEX based on GO is a common screening method [56]. Using the hydrophobic interaction with base, GO can adsorb ssDNA on surface by π–π stacking [57]. For example, Cho et al. [58] established a method for selecting aptamers by graphene oxide-adsorbed nanoparticles. The aptamer DNA01 of palladium ions was obtained with Kd of 4.6 ± 1.17 μM after 13 rounds of selection.

### Others

Nitrocellulose filter-SELEX is an in vitro aptamer selection method based on nitrocellulose filter with certain pore size [59]. The pore size of nitrocellulose filter can intercept the nucleic acid sequence that binds to the target, and the other nucleic acid sequence without binding to the target were separated [60]. The target is separated from the nucleic acid sequence by changing the nucleic acid solution environment, and then eluted from the nitrocellulose filter [61]. Tuerk and Gold [62] used nitrocellulose filter-SELEX method 30 years ago to select aptamers from T_4_ DNA phages and the aptamer quickly became a research hotspot. Kawakami et al. [63] selected aptamers with zinc in vitro, and then isolated and obtained Zn^2+^-dependent aptamers which novel novel RNA molecules in HIV-1Tat protein.

Circular dichroism (CD) is very sensitive to secondary structure of nucleic acid [64]. The spectral shape and signal of CD can effectively show the secondary structure changes of DNA caused by the binding to small molecules [65]. Isothermal titration calorimetry (ITC) is an intuitive and unlabeled method for analyzing and characterizing molecules which can directly measure the thermal changes caused by the chemical reactions between the two components [66, 67]. Therefore, CD and ITC can accurately monitor the binding between aptamer and target. Li and Ran [68] selected Cd^2+^ aptamer called issAP08-Cd^2+^with Kd of 2.9 μM via ITC. Moreover, Ran et al. [69] selected the Pb^2+^ aptamer with Kd of 1.13 μM via CD and ITC, which was named issAP17-Pb^2+^.

**Fig. 2:**
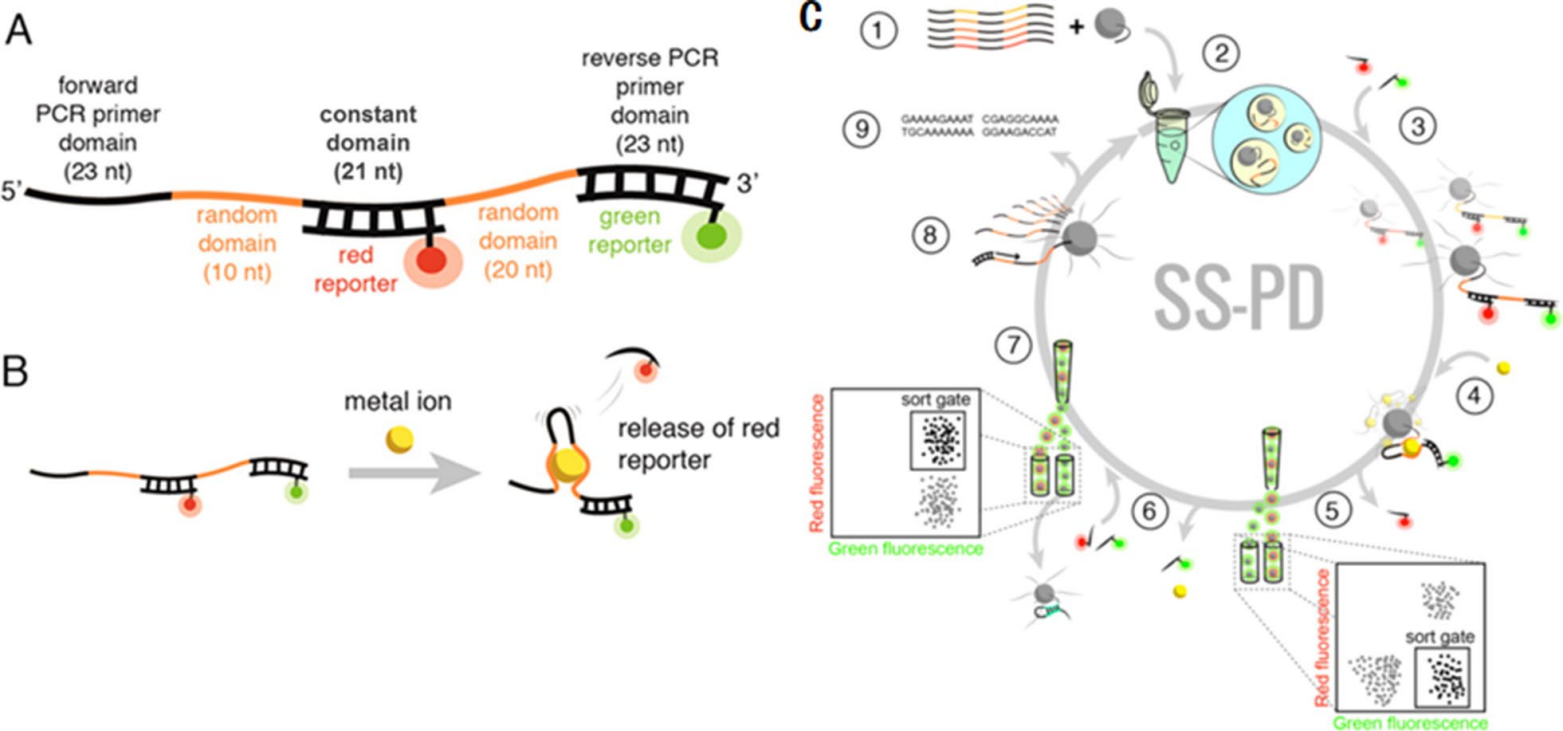
**A** Design of SS-PD aptamer library. **B** Heavy metal ions combine with aptamers causes the release of the red reporter. **C** The schematic diagram for selecting aptamers by SS-PD method [71]

Structure-switching particle display (SS-PD) is a novel method to produce high-quality structure-switching DNA aptamers in vitro [70]. When the sequence is bound to the target, the conformational change becomes large. The combination between each nucleic acid fragment and heavy metal ions is directly measured by fluorescence-activated cell sorting, and only the aptamers with the highest affinity are isolated. This method does not need any labeling or chemical modification, and the aptamers with high affinity can be separated in a short period of time. Qu et al. [71] used this method to obtain aptamers of Hg^2+^ and Cu^2+^ (Fig. 2).

Molecular dynamics (MD) simulation is a recently reported new aptamer selection method that can provide information such as orientation, folding-unfolding pathway and conformational rearrangement of aptamer, location of binding sites and availability of target sites [72]. This method modifies or mutates the existing sequence by examining the aptamer-target interaction to produce a new sequence with higher affinity. Khoshbin et al. [73] used this method to select the aptamers from Pb^2+^ and obtained the aptamer M4-16 with highest specificity.

The aptamers of heavy metals are difficult to screen, and the related reviews have not been reported. Table 1 shows the aptamer sequences of all heavy metals mentioned in this chapter.

**Table 1:** All the reports on the aptamers of heavy metal ions since the 21th century

Target	Name	Method	Cycle	Kd	Sequence	Refs
As^3+^	Ars-3	Affinity Chromatography	10	7.05 ± 0.91 nM	TTACA GAACA ACCAA CGTCG CTCCG GGTAC TTCTT CATCG	[54]
As^3+^	Ars-7	Affinity Chromatography	10	13.0 ± 1.25 nM	ATGCA AACCC TTAAG AAAGT GGTCG TCCAA AAAAC CATTG	[54]
Cd^2+^	Cd-4	Affinity Chromatography	11	34.5 nM	GGACT GTTGT GGTAT TATTT TTGGT TGTGC	[53]
Cd^2+^	CD-2–2	Affinity Chromatography	14	/	CTCAG GACGA CGGGT TCACA GTCCG TTGTC	[51]
Pb^2+^	Pb-14 s	Affinity Chromatography	18	0.76 ± 0.18 μM	GACGA CGGCC AGTAG CTGAC ATCAG TGTAC GATCT AGTCG TC	[52]
Pb^2+^	M4-16	molecular dynamics		/	GGGA GGGT GGGT GGGA	[73]
Zn^2+^	Zn-6m2	Affinity Chromatography	12	15 μM	GCATC AGTTA GTCAT TACGC TTACG GCCCG ATCCT AACTT GCTAC TGTCC CCTTC CGCCA GTTGT GCCGC GATTG TGAAG TCGTG TCCC	[50]
Co^2+^	NO.20	Affinity Chromatography	15	1.1 ± 0.15 mM	GGGCA UACGU UAGGC UGUAG GCGAG GUGGA AGAAA CGCGG UAAUA GCCUC AGCGU AGCAU AUGCA AGCUU CG	[55]
Pd^2+^	DNA 01	GO	13	4.6 ± 1.17 μM	GGGCG GACGC TAGGT GGTGA TGCTG TGCTA CACGT GTTGT	[58]
Hg^2+^	SSA-Hg II	SS-PD	4	2.27 ± 0.76 μM	TCCAA GCTCT TTTCT GCAGC TATTC TTGTT TCGAA ACTTG CTAAG CTGCG T	[71]
Cu^2+^	SSA-Cu II	SS-PD	4	47.15 ± 22.16 μM	ATCGC GATAT TTTCT GTAGC GATTC TTGTT TGAGC GCTCG GTACG AACAG A	

## Aptasensors for heavy metals ion detection

Compared with other targets, the simple structure and single binding site for heavy metal ions increase the difficulty for aptamer recognition [74]. The combination between heavy metal ions and aptamers will not occur with obvious mass change and steric hindrance effect [75]. This combination limits the application and sensitivity of many aptasensors. Although it is difficult to construct aptasensors for heavy metal ions detection, compared with traditional methods for detection of heavy metal ions, the aptasensor still has the advantages of high sensitivity and fast reaction speed [76]. This chapter introduces efficient methods for using aptamer sensora to detect heavy metal ions in the past five years.

### Optical aptasensors

Optical aptasensor is an important method for detection of heavy metal ions, including fluorescence aptasensor, colorimetric aptasensor, surface plasmon resonance (SPR) aptasensor, surface enhanced Raman scattering (SERS) aptasensor, fluorescence resonance energy transfer (FRET) aptasensor, chemiluminescence (CL) aptasensor and electrochemiluminescence (ECL) aptasensor [77–81]. In this chapter, the mechanism for the binding between aptamer and heavy metal ions is analyzed, and the application of above optical sensors to detect heavy metal ions is reviewed.

#### Lead

Lead is a heavy metal element with its ions (Pb^2+^) seriously endangering human health. The concentration of lead ions in the human body reaches a certain level, that it will cause cognitive and motor impairments, especially affecting the growth and intellectual development of children [82]. It was found in another study that Pb^2+^ can induce the folding of DNA structure [83]. Thrombin binding aptamer (TBA) is a ssDNA sequence rich in guanine, which contains 5′-GGTTGGTGTGGTTGG-3′ [84] chain. Under the action of Pb^2+^, TBA folds into a G-quadruplex structure. Many researchers fabricated aptasensors to detect Pb^2+^ using this aptamer. For example, a novel aptasensor based on FRET was reported for detection of Pb^2+^ in contaminated water by using Upconversion nanoparticles (UCNPs) as donor and gold nanoparticles (AuNPs) as acceptors [85]. The ssDNA sequence for the modified UCNPs was 5′-AAGGGTGGGTGGGT-3′, which could be folded into a G-quadruplex structure in the presence of Pb^2+^. When the conformation of aptamer was changed, the molecular distance between energy donor and acceptor also changed, and the green fluorescence for UCNPs was quenched. The concentration of Pb^2+^ was detected by fluorescence recovery, and the detection limit was as low as 4.1 nM. Colorimetric aptasensor can observe the color change through the naked eyes or spectrophotometer to realize the quantitative and qualitative detection of the target. Ouyang et al. [86] designed a SERS aptasensor based on gold nanoparticles (GNP) with nanoplasmonic SERS at 1614 cm^−1^. The Pb^2+^ aptamers were adsorbed on the surface of the AuNPs inhibited the its nonacatalysis and resulted in the decreased redox product for GNP nanoplasmonic effect. The aptamer formed a stable G-quadruplex in the presence of Pb^2+^ by binding to it, releasing free AuNPs and enhanced SERS signal. Tao et al. [87] developed a colorimetric aptasensor based on graphene/Fe3O4-AuNPs composites for monitoring Pb^2+^. The complementary aptamer chain competed with metal ions to bind aptamer. The aptamer bonded the target to form G-quadruplex when Pb^2+^ was added. Moreover, the free cDNA inhibited the catalytic activity adsorbed onto the surface of the graphene/Fe3O4-AuNPs composites. The aptasensor showed that absorbance of the reaction solution at 652 nm and concentration of Pb^2+^ had good linear correlation over the range of 1–300 ng/mL. Wu et al. [88] designed an aptasensor based on double fluorescent dye by using the same aptamer sequence. The synergistic application of two different dyes provided built-in correction for environmental conditions and improved the stability and accuracy while maintaining specificity. Ma et al. [89] also established a colorimetric aptasensor to detect trace lead ions by silver staining and Pb^2+^ aptamer of G-rich sequences [90].

The aptasensors for detection of Pb^2+^ using G-rich oligonucleotides as recognition elements have high selectivity, and researchers have improved the sensitivity of Pb^2+^ detection by amplifying the detection signal. For example, et al. [91] introduced an aptasensor based on quartz crystal microbalance (QCM), which amplified the frequency change by using oligonucleotide modified AuNPs to enhance its responsiveness. The aptamer immobilized on the surface of QCM combined with Pb^2+^ to prevent self-assembly of AuNPs on QCM. The trace concentration of QCM was determined by monitoring the change of Pb^2+^ response frequency. This sensor could detect Pb^2+^ in the range of 5-200 nM and detection limit was as low as 4 nM.

Chen et al. [52] in another study obtained the aptamer Pb-14S by SELEX method with good specificity and high affinity, and constructed a fluorescent aptasensor for detection of Pb^2+^ based on complex with Pb^2+^-induced release of fluorescence-labeled aptamer Pb-14S and a quencher-labeled short complementary sequence. The Pb-14S accurately identified Pb^2+^ and eliminated the interference of other metal ions with similar structure. The aptamer was transformed into stem-ring structure with addition of Pb^2+^ and the distance between fluorescent agent and quenching agent was changed, and the fluorescence intensity also changed. Moreover, the fluorescence intensity enhanced with Pb^2+^ concentration increased, which had a detection linear correlation in the range of 100 to 1000 nM. Aptamers with higher affinity need to be combined in future research with more effective signal amplification strategies to improve the sensor’s sensitivity.

#### Mercury

Mercury is one of the harmful water pollutions that can accumulate in the human body and damage the kidneys and capillaries [92, 93]. Kosturko et al. [94] proposed the crystal structure of 2:1 complex of 1-methylthymine-Hg^2+^ in 1974. This theory was put forward earlier than the concept of aptamer for 16 years, and it has become the most commonly used method for aptasensor based detection of Hg^2+^. Qi et al. [95] constructed a chemiluminescence aptasensor for the detection of Hg^2+^, where the recognition element was an aptamer with a sequence of 5′-TTT TTT TTT T-3′. Aptamer is a kind of polyanion that binds to ( +)AuNPs to inhibit the luminescence of H_2_O_2_ + Luminol system in the absence of Hg^2+^, which combines with aptamers to form T-Hg-T complex after addition of the target. ( +)AuNPs charge effect aptamer conformation change induced by Hg^2+^ effectively amplified luminescence of H_2_O_2_ + Luminol system. The low detection limit for this sensor to detect Hg^2+^ concentration was 16 pM. This group used the same Hg^2+^ aptamer and signal amplification strategy to design a colorimetric aptasensor with detection limit of 49 pM [96]. Wang et al. [97] moreover found that ssDNA bound a mixed-valence state cerium-based metal–organic framework (MVC-MOF), shielding its active sites and inhibiting its oxidase-like activity, while dsDNA activated its catalytic activity. In addition to Hg(II), the ion was bound to thymine-rich ssDNA (T-ssDNA) as a model DNA that caused formation of T-dsDNA if dsDNA was added. Hence, a colorimetric sensor to detect Hg^2+^ in water samples was designed. The aptamer bound with Hg^2+^ to produce heteroduplex and impaired the activity of mimicking the oxidase. The oxidase substrate 3,3′,5,5′-tetramethylbenzidine generates colored products under aerobic conditions, and the detection limit for Hg^2+^ was 10.5 nM. Wu et al. [98] designed an aptasensor based on G-quadruplex structure switching and T-Hg^2+^-T mismatch pair, and amplified the signal with two kinds of fluorescent dyes. Caglayan [99] applied SPR in another study to detect Hg^2+^ and made a SPR aptasensor with a detection limit of 26 pM.

Except for the self-folding of T-rich aptamers, T-Hg^2+^-T double-strands can be formed by artificially designing ssDNA that is partially complementary to aptamers. Sun et al. [100] for example introduced a fluorescent assay based on formation of T-Hg^2+^-T base pairs and aptamers-functionalized magnetic beads (AMB) for Hg^2+^. When the target was absent, the signal transduction probe (STP) could bind to the aptamer through complementary base pairing. After magnetic separation, the fluorescence signal was quite week owing to lack of STP in the detection solution. Help DNA competitively combined with AMB in presence of Hg^2+^, and caused formation of T-Hg-T base pairs. Moreover, STP was left in the detection solution after magnetic separation and fluorescence signal was significantly increased. The fluorescence intensity and Hg^2+^ concentration had good linear correlation in the range of 2–160 n and detection limit was 0.2 nM. Shi et al. [101] developed a FRET aptasensor based on T-Hg^2+^-T double-stranded structure. The aptamer-modified Copper @ Gold nanoclusters (apt-Cu@Au NCs) aggregated in the solution in the presence of Hg^2+^, resulting in change of fluorescence intensity. Determined by fluorescence analysis, the Hg^2+^ detection limit for the sensor was 4.92 nM.

#### Cadmium

**Fig. 3:**
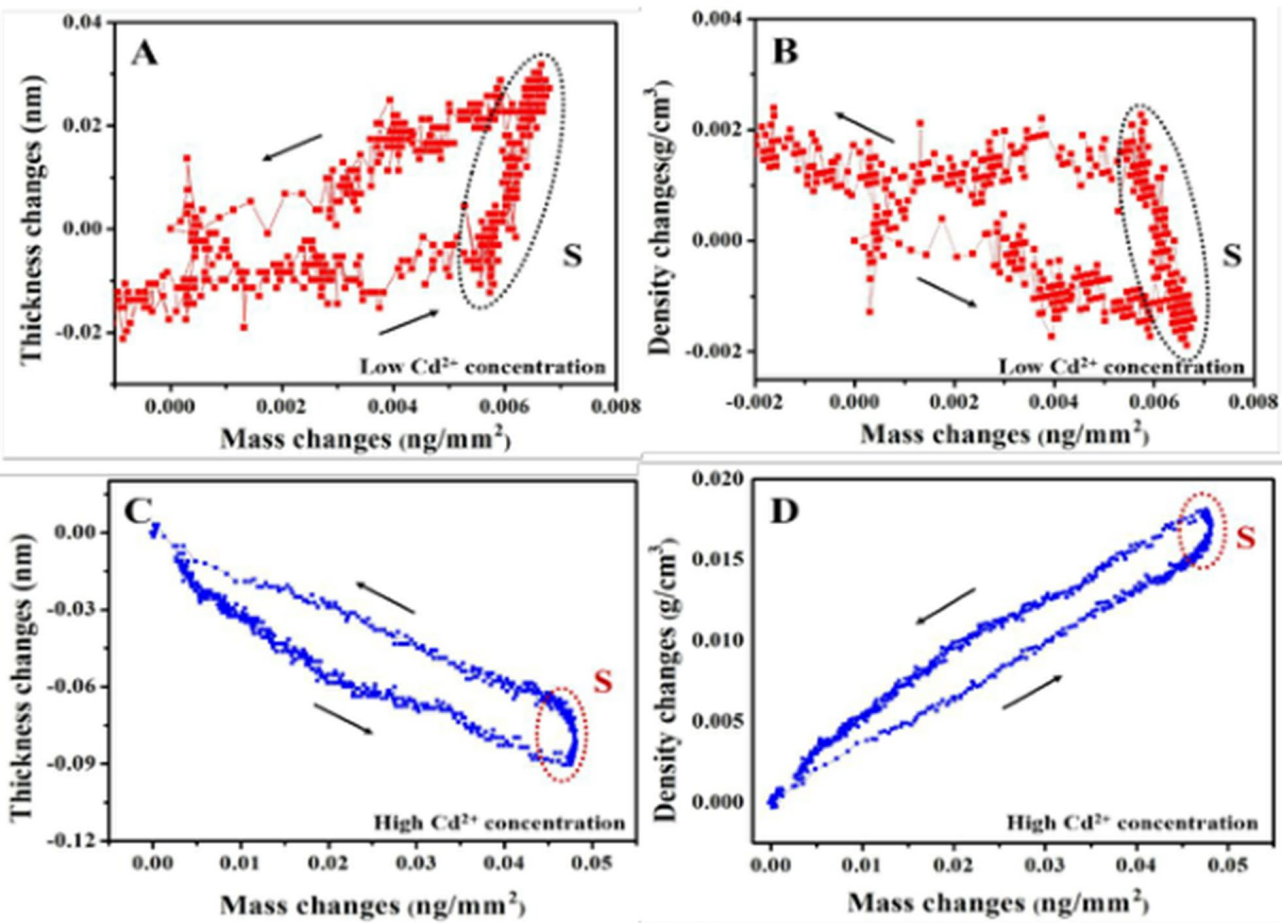
**A**, **B** At low concentration of Cd^2+^, the thickness increases and the density decreases with the increase of mass load. It is speculated that the binding of Cd^2+^ to aptamer is mainly through the binding of phosphate group. **C**, **D** At high concentration of Cd^2+^, the thickness decreases and density increases with increased load. It is speculated that Cd^2+^ leads to the conformational transformation of DNA through the π-π stacking of bases [104]

Cadmium has high toxicity. Food and water contaminated by cadmium do serious harm to the human body, and its metabolism is slow in the human body [102]. Cadmium accumulates in the human body, leading to the decline of lung function and renal failure. The development of simple and sensitive methods for rapid detection of cadmium ions (Cd^2+^) is therefore of significant importance [103]. In recent years, there have been many reports about Cd^2+^ aptasensors on detection of Cd^2+^. For example, Xue et al. [104] proposed the Cd^2+^ concentration-dependent interaction mechanism and conformation of aptamer by dual polarization interferometry (DPI) (Fig. 3). Under the action of low concentration of Cd^2+^, Cd^2+^ mainly interacted with phosphate groups from DNA to form extended ssDNA. The Cd^2+^ were bound with bases on the aptamer in the condition of high concentration of Cd^2+^, resulting in a tight and short hairpin structure through coordination interaction.

Gan et al. [105] designed a colorimetric sensor based on Cd-4 aptamer-functionalized AuNPs for Cd^2+^ detection. Aptamers could enhance the stability of AuNPs and avoid aggregation in high-salt solutions. The reason was that high-salt could shield the electrostatic repulsion among AuNPs and AuNPs aggregated when no aptamer was shrouded on the surface of AuNPs [106]. Free aptamers were reduced owing to the specific interaction between aptamers and Cd^2+^ when Cd^2+^ was added, which weakened the stability of AuNPs, resulting in AuNPs aggregation and strengthening ofsolution color. Zhu et al. [107] also designed a fluorescence sensor with a detection limit of 2.15 nM for Cd^2+^ by using Cd-4 aptamers. The 6-carboxyfluorescein (6-FAM) and GGGG sequences were modified at both ends of the Cd-4 aptamer, which could change the conformation after binding with Cd^2+^. G4 is close to 6-FAM and photoinduced electron transfer, which results in fluorescence quenching. Zeng et al. [108] reported a personal glucose meter based on Cd-4 aptamer for detection of Cd^2+^. The signal amplification process triggered specific recognition between the aptamer and Cd^2+^ by exonuclease III, which is an invertase conjugated complementary single-stranded DNA. The direct conversion was established between Cd^2+^ concentration and glucose amount by means of invertase conjugates hydrolyzing sucrose into glucose, and the sensor could detect Cd^2+^ at as low as 5 pM.

#### Other heavy metals

Arsenic is globally recognized carcinogen that is widely contaminated in groundwater due to the influence of human industry and mining [109]. As^3+^ is the most toxic form, with its toxicity about 60 times of As^5+^. Ars-3 aptamer for specific recognition of As^3+^ was obtained in 2009 and it’s for As^3+^ was 7.05 ± 0.91 nM. Ars-3 aptamer has been widely used in the construction of As^3+^ aptasensors. Siddiqui et al. [110] designed a colorimetric aptasensor based on aptamer Ars-3-AuNPs. This first reported device included extraction, purification and detection of As^3+^ from field soil. The extracted and purified samples effectively eliminated the effects of other interfering ions and organic acids. Ars-3 could effectively capture As^3+^ and induced AuNPs aggregation. The smartphone-based measurement system measured As^3+^ content in the field in only three hours, with detection limit of 1.97 ppm.

**Fig. 4:**
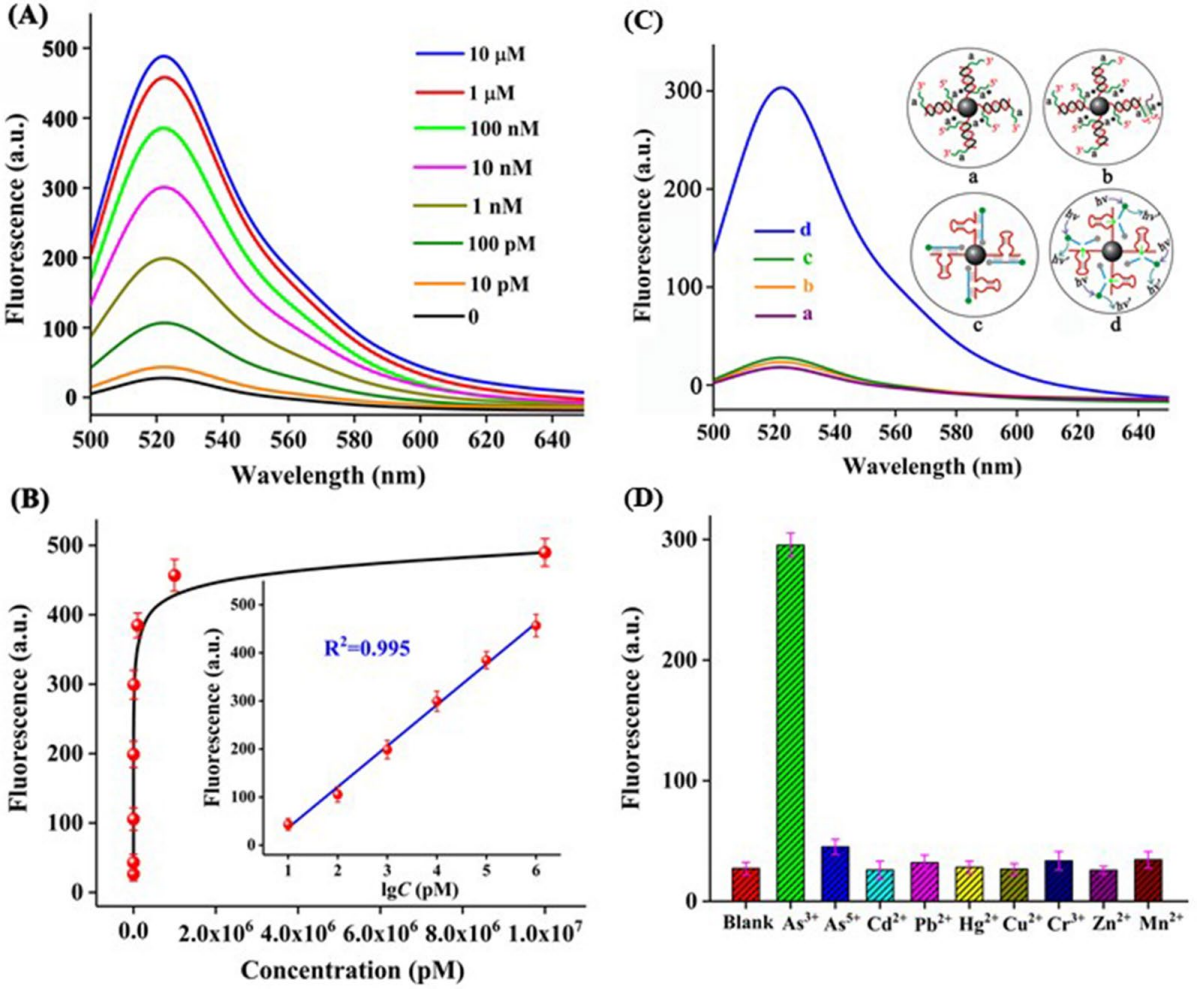
**A** The fluorescence intensity of the sensor at different As^3+^ concentrations. **B** The correlation between the logarithm of As^3+^ concentration and fluorescence intensity at 520 nm wavelength. **C** The fluorescence intensity of the sensor is: (a)without As^3+^, (b)without Exo III, (c)without Mg^2+^, (d)10 nM As^3+^, 2 unit/μL Exo III, and 100 mM Mg^2+^. **D** Selectivity investigation of sensing platform for As^3+^ against other competing heavy metal ions [112]

The aptamer sensor combined with effective signal amplification strategy can improve the sensitivity of the aptasensor. Nguyen et al. [111] studied a colorimetric aptasensor with improved sensitivity for detection of As^3+^ based on the synergistic molecular assembly of Ars-3 and cetyltrimethyl ammonium bromide (CTAB) on AuNPs. Using CTAB as binder could induce the aggregation of As^3+^ and Ars-3 aptamer-AuNPs, which resulted in visible color change. The detection limit for the prepared sensor was 16.9 ppb. Moreover, Zeng et al. [112] designed a fluorescence aptasensor based on continuous signal amplification strategy triggered by the target detection of As^3+^. The Ars-3 aptamer specially recognized As,^3+^ leading to the release of blocking DNA and triggering the subsequent signal amplification step. The sensor system produced a large number of Mg^2+^-dependent DNAzyme as the amplifier using Exonuclease III (Exo III)-mediated DNA cycle digestion. After magnetic separation, the active DNAzymes would continually catalyze and cleave substrate strands modified with fluorophore and quencher, thus a fluorescence signal for detection target was yielded and significantly amplified. The sensor system showed ultrasensitivity for detection of trace amount of As^3+^ owing to the synergetic signal amplification effect of EXO III and DNAzyme, and the detection limit was as low as 2 pM. Figure 4 demonstrates the response strength, correlation, signal amplification and specificity of the sensor to the target.

Apart from the widely used Ars-3, there are many reports on the use of other aptamers to construct As^3+^detection sensors in recent years. Zhang et al. [113] established a SPR aptasensor for the determination of arsenic ion based on excellent catalytic properties of Au-doped carbon dots (CD_Au_) and high specificity of Ars-7 aptamer, which is reported by Kim. CD_Au_ could effectively catalyze the nano-reaction of AgNO_3_ reduction by glucose, which produced yellow silver nanosal with a strong SPR absorption peak at 420 nm. Ars-7 aptamer could inhibit the catalytic activity of CD_Au_, leading to change of color of the solution to become lighter. After As^3+^ was added, it was bound to the aptamer and released free carbon dots. The catalytic activity of CD_Au_ was therefore restored, resulting in linear enhancement of solution color and Abs signals. The linear relationship for SPR spectrophotometric assay was in the range of 0.025–0.75 μg/L, with detection limit of 0.01 μg/L for the determination of As^3+^.

**Fig. 5:**
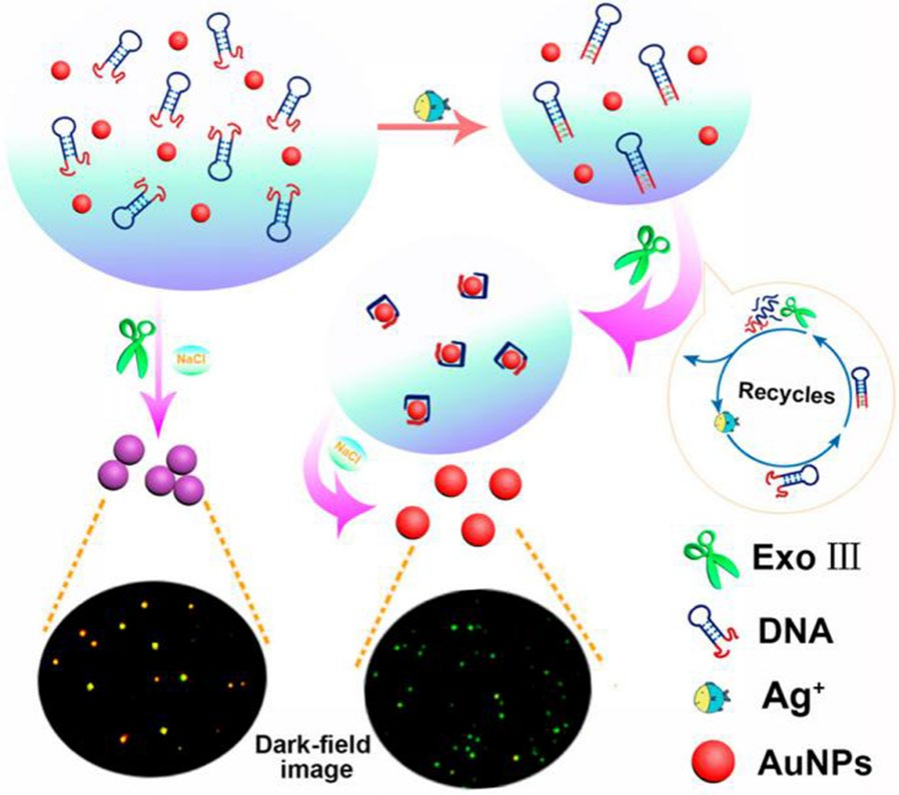
The interaction between aptamer and Ag^+^ can be detected by dark field microscope under the action of Exo III dependent DNA cleavage cycle amplification. [114]

Similar to T-Hg^2+^-T, Ag^+^ can form C–Ag^+^–C mismatch with cytosine. Via this characteristic, the sensitive aptamer sensor can be designed for Ag^+^ detection. For example, Li et al. [114] designed an aptamer sequence that could form a hairpin structure with Ag^+^, and established a colorimetric sensor based on Ag^+^ and Exo III-dependent DNA cleavage recycling amplification (Fig. 5). In this sensor, Exo III could cut the dsDNA carrying Ag^+^, and the released ssDNA was adsorbed on the surface of AuNPs. The strong electrostatic repulsion among the AuNPs therefore promoted the dispersion of AuNPs in high salt media, and the color changed to green (disperse AuNPs) under dark-field microscopy observation. On this basis, the detection limit for the Ag^+^ sensor was as low as 41 fM.

#### Multi-target detection

In the actual sample detection, many heavy metal ions generally exist in different biological and environmental systems [115]. The heavy metal ions pollution produced by the industrial production process will enter the human body through water and food. Besides, the sensor for detecting a single target is easily disturbed by other heavy metal ions. Therefore, it is of great significance to develop a method that can detect many types of heavy metal ions at the same time. For example, Khoshbin et al. [116] designed a fluorescent aptasensor that could simultaneously detect Hg^2+^ and Ag^+^ in about 10 min. This sensor contained specific identification Hg^2+^ and Ag^+^ aptamers that were modified by FAM and fixed on different regions of GO. The aptamers can be eluted from GO by target and recover the fluorescence signal suppressed by GO. Via monitoring of fluorescence recovery rate to determine the ion concentration, the detection limit for Hg^2+^ and Ag^+^ were as low as 1.33 pM and 1.01 pM, respectively. Similar to the principle of above sensor, Lu et al. [117] constructed a fluorescent aptasensor based on GO and fluorescently labeled aptamer probes for simultaneous detection of Pb^2+^, Hg^2+^, and Ag^+^. In general, these free single-stranded probes were adsorded on the surface of GO. Owing to the close proximity between fluorophores and GO, the fluorescence resonance energy transfer occurred, resulting in fluorescence quenching and signals weakening. The fluorescence intensity was restored after the target ions were added. The fluorescence intensities for the three fluorophores and corresponding ions concentration had good linear dependence. Besides this prepared sensor, Lu et al. [118] also developed a fluorescent sensor that could detect Pb^2+^ and Hg^2+^ at the same time. The sensor had two kinds of quenchers which could suppress the fluorescence signal under general conditions. The ions bonded with the aptamer and destroyed its stem-loop structure after addition of the target, and thus the fluorescence signal was restored. The average recovery in the actual sample was 96.55–102.78%, which proved that this method had high accuracy. Moreover, Feng and Li [119] developed an electrochemiluminescence (ECL) sensor for simultaneous detection of Pb^2+^ and Hg^2+^. The Pb^2+^ and Hg^2+^ aptamers combined with targets to form G-quadruple and hairpin structure, resulting in ECL intensity difference. The sensitivities of the sensor for detecting Pb^2+^ and Hg^2+^ were 24 pM and 4.1 pM, respectively. Based on regulation of oxidase-mimicking activity of Mn_3_O_4_ nanoparticles (Mn_3_O_4_NPs) by oligonucleotides, Wang et al. [120] established a colorimetric method for the detection of Hg^2+^ and Cd^2+^. With combination of target and aptamer, TMB changed from light green to yellow under Mn_3_O_4_NPs catalysis.

Optical aptasensors have been widely used because of its simple preparation and intuitive results. Table 2 integrates the application of aptasensor in the detection of heavy metal ions in recent five years.

**Table 2:** A summary of developments of optical aptasensor for heavy material ions

Target	Type	LOD (nM)	LinearRange (nM)	Aptamer	Refs
Pb^2+^	FRET	4.1	0–50	AAGGG TGGGT GGGT	[85]
Pb^2+^	SERS	0.07	0.13–53.33	GGTTG GTGTG GTGGT TGGTG TTGG	[86]
Pb^2+^	Colorimetric	3.04	4.83–1447.88	GGGTG GGTGG GTGGG T	[87]
Pb^2+^	Colorimetric	100	500–10,000	GTGGG TAGGG CGGGT TGG	[89]
Pb^2+^	QCM	4	5–200	TTTTT TACCC AGGGT GGGTG GGTGG GT	[91]
Pb^2+^	Fluorescence	60.7	100–1000	GACGA CGGCC AGTAG CTGAC ATCAG TGTAC GATCT AGTCG TC	[52]
Hg^+^	Colorimetric	0.049	0.82–62	TTTTT TTTTT	[96]
Hg^+^	Colorimetric	10.5	50–6000	TTTTT TTTTT TTTTT TTTTT TTT	[97]
Hg^+^	SPR	0.026	0.05–100	TTTTT ATTCT TTCTT CCCCC CGGTT GTTTG TTT	[99]
Hg^+^	Fluorescence	0.2	2–160	TTCTT TCTTC TTTC	[100]
Hg^+^	FRET	4.92	500–7000	AATAG CTTTG CTCTC TCGTT T	[101]
Cd^2+^	Colorimetric	9.96	17.79–177.92	ACCGA CCGTG CTGGA CTCTG GACTG TTGTG GTATT ATTTT TGGTT GTGCA GTATG AGCGA GCGTT GCG	[105]
Cd^2+^	Fluorescence	2.15	7.19–5000	GGGGA CTGTT GTGGT ATTAT TTTTG GTTGT GCAGT	[107]
Cd^2+^	Colorimetric	5 × 10^–3^	0.02–2 × 10^5^	ACCGACCGTGCTGGACTCTGGACTGTTGTGGTA TTATTTTTGGTTGTGCAGTATGAGCGAGCGTTGCG	[108]
As^3+^	Colorimetric	185	/	GGTAA TACGA CTCAC TATAG GGAGA TACCA GCTTA TTCAA TTTAA GAACA ACCAA CGTCG CTCCG GGTAC TTCTT CATCA GATAG TAAGC AATCT	[110]
As^3+^	Colorimetric	217	12.8–1282.9	GGTAA TACGC TCACT ATAGG GAGAT ACCAG CTTAT TCAAT TTTAC AGAAC AACCA ACGTC GCTCC GGGTA CTTCT TCATC GAGAT AGTAA GTGCA ATCT	[111]
As^3+^	Fluorescence	2 × 10^–3^	0.001–1000	GGTAA TACGA CTCAC TATAG GGAGA TACCA GCTTA TTCAA TTTTA CAGAA CAACC AACGT CGCTC CGGGT ACTTC TTCAT CGAGA TAGTA AGTGC AATCT	[112]
As^3+^	Colorimetric	0.128	0.32–9.62	ATGCA AACCC TTAAG AAAGT GGTCG TCCAA AAAAC CATTG	[113]
Ag^+^	Colorimetric	4.1 × 10^–5^	5.7 × 10^–5^–0.57	CCCCC CCGTG GGTAG GGCGG GTTGG ACCCT ACCCA CCCCC CCG	[114]
Ag^+^; Hg^2+^	Fluorescence	1.01 × 10^–3^; 1.33 × 10^–3^	0.05–50; 0.05–50	CCCCC CCCCC CC; TTTTT TTTTT TT	[116]
Hg^2+^; Pb^2+^; Ag^+^	Fluorescence	0.2; 0.5; 2	0.3–14; 0.8–38; 4.2–210	TTCTT TCTTA ACTTG TTTGT TCAC; GGAAG GTGTG GAAGG AAC; CTCTC TTCTC TTCAT AAATC AACAC AACAC ACAAA	[117]
Pb^2+^; Hg^2+^	ECL	0.024; 0.0041	0.1–10^4^; 0.01–100	AAAAA AAAAG GGG TTTTT TAAAA TTTTT T	[119]

### Electrochemical aptasensors

Electrochemical aptasensor consists of electrodes and recognition molecules with electrochemical activity. After the aptamer fixed on the electrode surface combines with the target, the concentration of the target is detected by collecting the changes of electrochemical signals such as voltage, current, conductivity and impedance [121]. Common electrochemical methods include sandwich method, competition method and electrochemical impedance spectroscopy method [122, 123]. In addition, electrochemical sensors can also be combined with electrochemical signal amplification strategy to achieve the purpose of amplifying the signal. Electrochemical sensors are gradually favored by researchers because of their characteristics, such as multiple analysis, fast detection speed, high sensitivity and relatively low cost [124, 125]. Table 3 integrates the application of electrochemical aptasensor in the detection of heavy metal ions in recent 5 years.

**Table 3:** A summary of developments of electrochemical aptasensor for heavy material ions

Target	nanomaterial	LOD (nM)	LinearRange (nM)	Aptamer	Refs
Hg^2+^	AuNPs/CS	5 × 10^–3^	0.01–500	TCATG TTTGT TTGTT GGCCC CCCTT CTTTC TTA	[127]
Hg^2+^	AuNPs	1 × 10^–4^	2 × 10^–3^−20	TTCTC TCTTC GACGT TGTGT GTT	[128]
Hg^2+^	NFs-QDs	0.02	0.1–150	TTTTT TTTTT ACAGC AGATC AGTCT ATCTT CTCCT GATGG GTTCCT ATTTA TAGGT GAAGC TGT	[153]
Hg^2+^	AgNPs	25	25–500	TTTCT TCTTT CTTCC CCCCT TGTTT GTTT	[130]
Pb^2+^	ERGO	5.1 × 10^–7^	10^–6^−1	GGTGG TGGTG GTTGT GGTGG TGGTG G	[132]
Pb^2+^	Au@Py	2.9	2.4–120.7	GGGTG GGTGG GTGGG T	[133]
Pb^2+^	AuNPs	8.5 × 10^–3^	0.01–1000	GGGTG GGTGG GTGGG TGGGT	[134]
Pb^2+^	PtNPs@PCs	0.018	0.05–1000	GGGTG GGTGG GTGGG TAT	[135]
Cd^2+^	rGO/g-C_3_N_4_	0.337	1–1000	C_12_-GGGGC AGTGC CTCAC AACCT	[136]
As^3+^	CNPs	1.18	6.4–1283	GGTAA TACGA CTCAC TATAG GGAGA TACCA GCTTA TTCAA TTTTA CAGAA CAACC AACGT CGCTC CGGGT ACTTC TTCAT CGAGA TAGTA AGTGC AATCT	[137]
As^3+^	Ag-Au alloy NPs	3.8 × 10^–5^	0.128–128	GGTAA TACGA CTCAC TAAGG GAGAT ACCAG CTTAT TCAAT TTTAC AGAAC ACCAA CGTCG CTCGG GTACT TCTTC ATCGA GATAG TAAGT GCAAT CT	[138]
As^3+^	/	0.26	1.28–2565.7	TGATGTTTGTTTACGCATGTGTGAGGAGAGGCTGGGGTGATGAATCCCAATCCC	[139]
Pb^2+^; As^3+^	Fe-MOF@mFe_3_O_4_@mC	2.27 × 10^–3^ 6.73 × 10^–3^	0.01–10; 0.01–10	CAACG GTGGG TGTGG TTGG; GGTAA TACGA CTCAC TATAG GGAGA TACCA GCTTA TTCAA TTTTA CAGAA CAACC AACGT CGCTC CGGGT ACTTC TTCAT CGAGA TAGTA AGTGC AATCT	[141]
Pb^2+^; Hg^2+^	/	0.48; 0.50	0.48–4826; 0.50–4985	CAACG GTTGG TGTGG TTGG; TTCTT TCTTC CCCTT CTTTC TT	[143]

Generally, labeled electrochemical aptasensor is via labeling some functional markers with electroactive or catalytic activity by adsorption and modification at the end of the aptamer probe. The common functional markers are methylene blue (MB) and ferrocene (FC). The combination of aptamer and target leads to increased or decreased electrochemically active substances and subsequent change of electrochemical signals. The aptamer probe for the label-free electrochemical aptasensor can directly monitor the interaction between aptamer and target without the labeling of the electroactive material [126]. Electrochemical impedance spectroscopy (EIS) is the most common method for label-free electrochemical aptasensors, which is particularly sensitive in detecting the conjugation reaction on the surface of the electrode.

#### Mercury

Hg^2+^ is a widely used target for detecting heavy metal ions by electrochemical aptasensors. T-Hg^2+^-T is the recognition principle of electrochemical sensor for Hg^2+^ detection. Liu et al. [127] established a mercury ion electrochemical sensor based on T-Hg^2+^-T structure. The aptamer-functionalized AuNPs and chitosan were modified on the glassy carbon electrode (aptamer(AuNPs/CS)_2_/GCE) because AuNPs could enhance the electron transfer and improve analytical response. The aptamer recognized the Hg^2+^ and folded into the hairpin structure, resulting in the electrochemical signal indicator close to the surface of the sensor. Thus, an electrochemical response was produced. Based on this mechanism for the biosensor, the logarithm of Hg^2+^ concentration and DPV peak current had a good linear relationship in the range of 0.01–500 nM, and the detection limit for the prepared sensor was 5 pM. Si and Tang [128] developed the electrochemical sensor for determination of mercury ions based on specificity of thymine-rich Hg^2+^ aptamer and high catalytic activity of template deoxynucleotidyl transferase (TdT). Template-independent TdT improved the sensitivity of sensor by lengthening the aptamer end of 3'-OH by repeating bases, and Hg^2+^ aptamer brought selective interaction of thymine mismatched pairs, enhancing the specificity. Based on this strategy, the detection limit for the sensor was 0.1 pM. Moreover, a combination based on electro spun nanofibers polyethersulfone and quantum dots (NFs-QDs) was designed in another study for sensitive detection of Hg^2+^ [129]. In this sensor, the electrochemical signal of MB was amplified by NFs-QDs nanocomposites after the aptamer was bound to Hg^2+^. In another research on impedance electrochemical sensors, ink-jet printed and aptamer functionalized gold electrodes were proposed for the first time as a reliable, stable and fully scalable method for the detection of Hg^2+^ in water and organic solvents [130, 131]. The results demonstrated that the aptasensor had good stability and remarkable repeatability under harsh conditions. The sensor could detect the Hg^2+^ of 0.005 ppm in organic solvents.

#### Lead

In recent years, electrochemical aptasensors for Pb^2+^ detection are mainly based on the capture of Pb^2+^ by G-quadruplex. Under the same principle of recognition target, DNA sequence can realize the specific recognition of Pb^2+^. However, the key to developing a highly sensitive sensor is to fix the nanomaterials on the aptamer. For example, Yu et al. [132] established an ultra-sensitive electrochemical sensor for the detection of Pb^2+^ based on G-quadruplex aptamer and electrochemical reduction of graphene oxide (ERGO) electrode. Methylene blue (MB)-labeled G-rich aptamers were immobilized on the ERGO-modified glassy carbon electrode (GCE) by π-π interaction. When Pb^2+^ was added, the aptamer specifically recognized Pb^2+^ induced allosteric G-quadruplexes and detached from the ERGO surface, and then the redox current labeled with MB changed significantly. The sensor detection limit for Pb^2+^ was 0.51 fM. Based on the Pb^2+^ aptamer, Au nanoparticles composites and polypyrrole (Au@Py), Ding et al. [133] established an electrochemical sensor for detection of Pb^2+^.The AuNPs of Au@Py composites could increase signal transduction and amplify the current signal when Au@Py modified on the surface of screen-printed electrode. Toluidine blue (TB) for biosensor is a redox indicator that can interact with dsDNA consisting of Pb^2+^ aptamer and complementary strand of the aptamer. After adding the target Pb^2+^, the peak current of the interaction between TB and dsDNA was lower than that without target for the combination of the aptamer and Pb^2+^.

Tang and colleagues [134] designed an electrochemical sensor based on label-free G-quadruplex for the detection Pb^2+^. Polyion oligonucleotide-labeled gold nanoparticles modified on the gold electrode, as signal-amplified probes, provided high-density negative charge on the electrode surface. The Pb^2+^-induced G-quadruplex formation reduced the negative charge on the surface of the electrode in the presence of Pb^2+^, realizing the highly selective detection of Pb^2+^. The sensitive aptasensor exhibited wide detection range from 10^–11^ to 10^–6^ M and the detection limit of 8.5 pM. In another report on detection of Pb^2+^ by label-free electrochemical sensor, Jin et al. [135] adopted Pb^2+^ aptamer-based catalytic hairpin assembly technology and composite of platinum nanoparticles (PtNPs)/porous carbon(PCs) (PtNPs@PCs) catalyzed hydroquinone-H_2_O_2_ system as signal amplification strategy. Their results showed that the electrochemical signal depended on the concentration of Pb^2+^ in the range of 50–1000 nM.

#### Cadmium

**Fig. 6:**
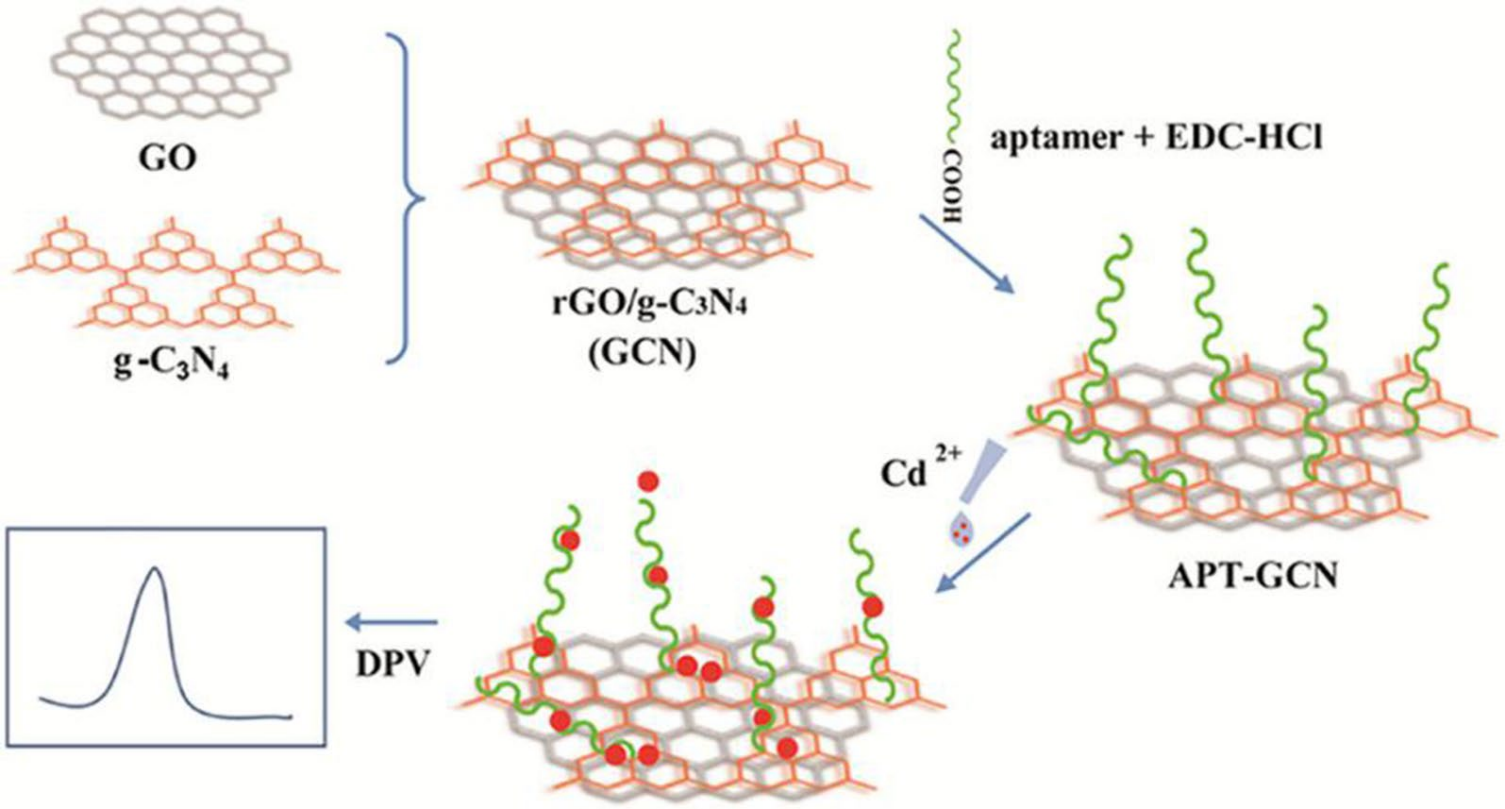
Schematic illustration of the fabrication of GCN aptasensor. The sensor can produce obvious DPASV signal change when detecting Cd^2+^ [136]

There are a few reports on electrochemical aptamer sensors to detect Cd^2+^ in recent years. After the IssAP08-Cd^2+^ aptamer was selected, it was used to prepare a label-free electrochemical aptasensor to recognize Cd^2+^ [68]. The aptamer was modified on the gold screen printed electrode. The conformational change of aptamer led to the change of surface impedance in the presence of Cd^2+^, and current density signal. Based on the reduced graphene oxide (rGO)/graphite carbon nitride (g-C_3_N_4_) composite system (GCN), Wang et al. [136] constructed an aptasensor with a detection limit of 0.337 nM for Cd^2+^ (Fig. 6). The rGO could improve the electrical conductivity, and concentration of Cd^2+^ in the solution was monitored by differential anodic stripping voltammetry (DPASV).

#### Other electrochemical sensors

After the As^3+^ aptamer was reported, there were many reports on electrochemical aptasensor for the detection of As^3+^. For example, Mushiana et al. [137] reported an electrochemical aptasensor based on aptamer Ars-3 and carbon and gold nano-platform (CNPs/AuNPs). A thiol modified Ars-3 aptamer was immobilized on GCE modified by CNPs/AuNPs via Au–S bond. As a signal amplifier, CNPs could effectively amplify the electrochemical signal of Ars-3 combined with As^3+^ on the surface of AuNPs. The detection limit for the prepared sensor was 0.092 ppb. Yadav and his colleagues [138] reported an electrochemical aptasensor based on aptamers and Ag–Au alloy nanomaterials (Ag–Au alloy NPs) to detect As^3+^. The sensor was used As^3+^ aptamer deep trapped Ag-Au alloy NPs which modified glassy carbon electrode. The captured As^3+^ started the electrochemical reaction, which generated a significant increase in the current of voltammetry curves. The detection limit for the As^3+^ sensor was 3 pg/L. Moreover, Gu et al. [139] established an electrochemical sensor for the detection of As^3+^ based on intercoordination between hybridization chain reaction (HCR) and RecJ_f_ exonuclease (RECJ_f_ exo) catalyzed reaction. The aptamer and ssDNA were assembled on the surface of the gold electrode, resulting in a huge charge transfer resistance (R_ct_). The aptamer sequence was specifically bound to As^3+^ in the presence of As^3+^ and DNA was dissociated. The release of HCR products could reduce R_ct_, which RECJ_f_ exo could further amplify this signal. The detection limit for this sensor was as low as 0.02 ppb.

**Fig. 7:**
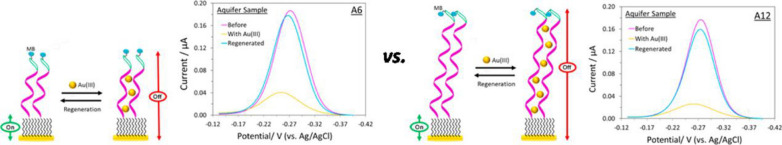
The signal mechanism of six adenines (A6) or twelve adenines (A12) were used as DNA probes to construct the signal mechanism of the sensor. The combination of the target and the probe can harden the probe and reduce the MB current [140]

According to the study on binding mechanism for Au and DNA, it was found that, polyA DNA could be tightly adsorbed on the surface of Au, while the adsorption of polyT DNA was weak. Wu et al. [140] reported an electrochemical ion (E-ion) sensor to detect Au^3+^. Based on the interaction between Au^3+^ and adenine, MB as a signal switch, was modified at the end of an adenine-rich ssDNA. The complex formed between Au^3+^ and ssDNA hardened the probe, and MB was limited to enter the electrode and reduced the signal intensity (Fig. 7).

#### Multi-target detection

The detection of multi-target also uses the recognition effect of aptamer to the target, and the aptamers from many types of ions are fixed on the electrode. For example, Zhang et al. [141] designed new type of core–shell nanostructured composites, which could provide a large number of aptamer binding sites. The composites consisted of Fe (III)-based metal–organic framework (Fe-MOF) and mesoporous Fe_3_O_4_@C nanocapsules. Via conformational transition interaction between aptamer and target, the target ions would block and obstruct the surface of the nanocomposite, causing decreased electron transfer, and further leading to the increased *R*_*ct*_. The detection limits for the Pb^2+^ and As^3+^ sensor were 2.27 and 6.73 pM, respectively.

**Fig. 8:**
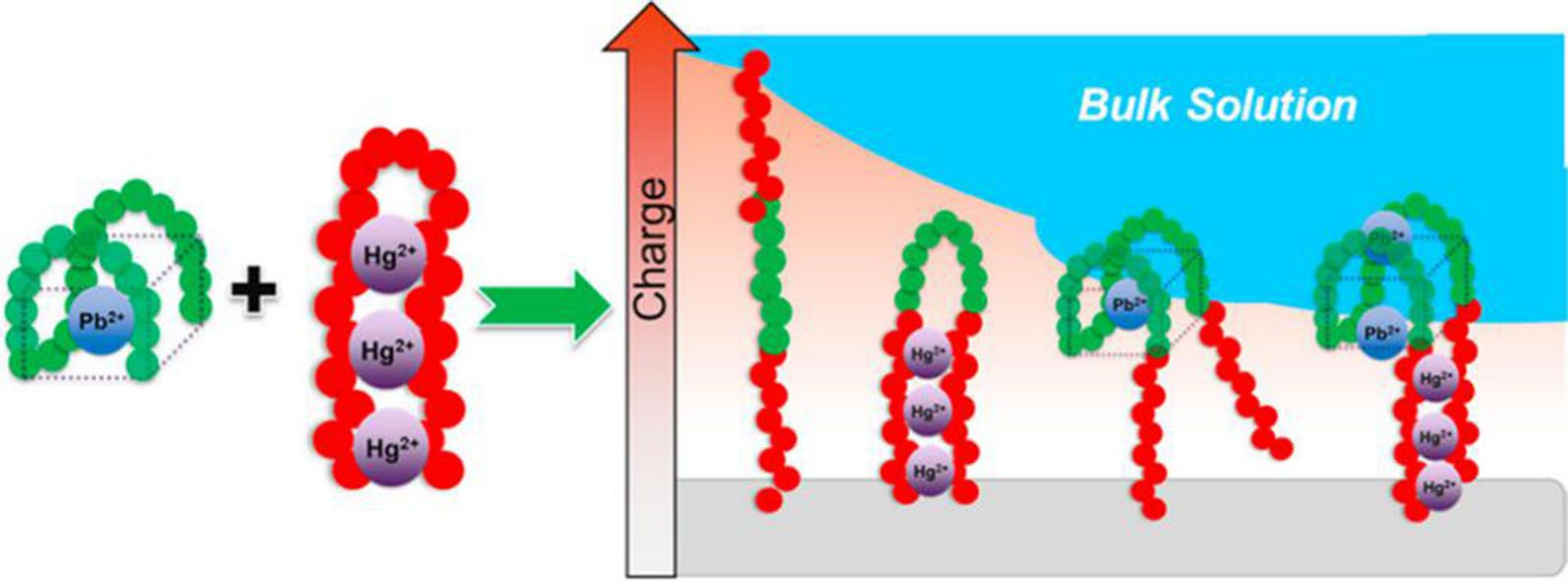
Schematic diagram of double ligand binding target in multi-target aptamer sensor [142]

Based on aptamer recognition and resistive pulse (RP) sensor technology, Mayne et al. [142] prepared an aptamer sensor for monitoring of Pb^2+^ and Hg^2+^. RP had single particle resolution, which allowed the sample to be characterized. When the nanomaterials that were modified with aptamers passed through the RP sensor, the information such as charge state and density could be observed. The combination of targets led to decreased charge density, then obtaining the target concentration information. The sensor carried different aptamers to adapt to detect different targets. Figure 8 shows the binding principle of multi-target aptamers.

In another report on the detection of Pb^2+^ and Hg^2+^, a sensor with a detection limit of 0.1 ppb was constructed [143]. In this sensor, the FC modified by aptamer was fixed on the screen-printed gold electrode to detect the target concentration by monitoring the electron transfer between the redox label and the electrodes.

### Other aptasensors

In recent years, the aptamer sensors for detecting heavy metal ions have made great progress in their research [144–146]. The challenges of aptasensors, including specificity, sensitivity and rapid response are constantly innovating. In addition, some novel aptasensors have been reported in recent years. For example, some Field-Effect Transitor (FET)-based aptasensor was introduced. Wang et al. [147, 148] constructed an FET sensor based on aptamer and single-walled carbon nanotubes (SWNTs) to detect Pb^2+^. The double-stranded DNA containing aptamers was coupled to SWNTs. The combination of Pb^2+^ and aptamer led to deionization, which affected the electrical conductivity of SWNTs as an analytical signal. The sensor detected Pb^2+^ concentration as low as 0.39 ng/L. Due to its anisotropic properties and molecular sensitivity, liquid crystals (LCs) have been used for detecting chemical and biological events by converting chemical and biological intereactions into optical signals under a polarized light microscope. Nguyen et al. [149] reported a sensor based on As^3+^ aptamer Ars-3 and label-free LCs. Cetyltrimethylammonium bromide (CTAB) installed independently at the aqueous/LC interface could induce homeotropic orientation of LCs. The negatively charged aptamers interfered with self-assembly of positively charged CTAB on the aqueous/LC interface. The orientational of LCs was changed into planar state without As^3+^. The addition of As^3+^ could weaken the interference of aptamer to CTAB and make LC keep in a homeotropic state. The detection limit for this sensor was 50 nM.

In addition, Ertan et al. [150] established an ellipsometric aptasensor to detect Hg^2+^. Chen et al. [151] established a luminescence resonance energy transfer (LRET) sensor for detecting As^3+^. Liu et al. [152] established a sensor that can detect Hg^2+^ based on the attenuated total reflection surface enhanced infrared absorption spectroscopy (ATR-SEIRAS).

## Conclusion and future prospect

In the past three decades, aptamers have been widely used in disease diagnosis, targeted drug delivery, environmental detection, pesticide residues and other fields, because of their excellent properties, such as easy modification, low cost and strong specificity. In order to detect heavy metal ions by aptasensor, two problems should be solved. First, heavy metal ions have the characteristics of simple conformation and single binding site, so it is difficult to obtain aptamers with high specificity and high affinity. Secondly, due to the small molecular weight of the target, it is necessary to overcome the huge steric hindrance when combining with the aptamer, which poses a great challenge to the sensitivity of the sensor. The aptamers of heavy metal ions mainly include Pb^2+^, Hg^2+^, Cd^2+^ and As^3+^, which are heavily polluted from industry. Aptasensors have therefore become an important tool to detect heavy metal pollution. Some sensors detect the concentration of heavy metal ions to as low as fM concentration.

Although aptasensors have made great achievements in the detection of heavy metal ions in the past few decades, there are still many challenges. First, the reports on the selection of aptamers for heavy metal ions are mainly focused on the common ions for industrial pollution, while the aptamers from other heavy metal ions are rarely reported. Even many heavy metal ions have not obtained aptamers with high affinity and strong specificity. Secondly, the sensor with higher sensitivity has higher requirements for sample pretreatment, laboratory equipment and professional operators, so it is difficult to achieve on-site detection. Third, the construction of the sensor is difficult, and the stability and toxicity of nano-materials will limit the application of the aptasensor.

In order to solve these problems, further research on aptasensors to detect heavy metal ions should pay attention to the diversity of targets and commercialization of sensors. In order to deal with detection of various targets, the future development of the sensor should detect several heavy metal ions in one platform. The aptamer with high affinity and detection methods with high sensitivity can be designed as a sensitive aptasensor for heavy metal ions detection. At present, aptasensors mostly rely on laboratory instruments and professional operators during the detection of actual samples, it is therefore easy to be affected by other ions or organic compounds, which often requires complex pretreatment steps. All these measures will enable the aptamer sensor to achieve rapid and sensitive on-site detection. In summary, we expect the aptasensors to become an effective analytical tool for the detection of heavy metal ions to meet various challenges.

## Data Availability

Not applicable.

## Abbreviations

SELEX: Systematic evolution of ligands by exponential enrichment
NPs: Nanoparticles
CD: Circular dichroism
GO: Graphene oxide
ITC: Isothermal titration calorimetry
SS-PD: Structure-switching particle display
MD: Molecular dynamics
SPR: Surface plasmon resonance
SERS: Surface enhanced Raman scattering
FRET: Fluorescence resonance energy transfer
CL: Chemiluminescence
ECL: Electrochemiluminescence
TBA: Thrombin binding aptamer
UCNPs: Upconversion nanoparticles
AuNPs: Gold nanoparticles
GNP: Gold nanoparticles
QCM: Quartz crystal microbalance
T-ssDNA: Thymine-rich ssDNA
AMB: Aptamers-functionalized magnetic beads
STP: Signal transduction probe
DPI: Dual polarization interferometry
6-FAM: 6-Carboxyfluorescein
CTAB: Cetyltrimethyl ammonium bromide
CD_Au_: Au-doped carbon dots
MB: Methylene blue
FC: Ferrocene
EIS: Electrochemical impedance spectroscopy
TdT: Template deoxynucleotidyl transferase
NFs-QDs: Nanofibers polyethersulfone and quantum dots
ERGO: Electrochemical reduction of graphene oxide
GCE: Glassy carbon electrode
TB: Toluidine blue
DPASV: Differential anodic stripping voltammetry
HCR: Hybridization chain reaction
Fe-MOF: Fe (III)-based metal–organic framework
SWNTs: Single-walled carbon nanotubes
CTAB: Cetyltrimethylammonium bromide
